# Clinico-Microbiological Correlates of Hospital-Acquired Pneumonia: A Hospital-Based Prospective Cohort Study

**DOI:** 10.7759/cureus.50707

**Published:** 2023-12-18

**Authors:** Avisham, Anurag Agrawal, Abhishek Gupta

**Affiliations:** 1 Department of Respiratory Medicine, Government Doon Medical College, Dehradun, IND; 2 Department of General Medicine, Himalayan Hospital, Swami Rama Himalayan University, Dehradun, IND; 3 Department of General Medicine, Government Doon Medical College, Dehradun, IND

**Keywords:** hospital-acquired pneumonia, ventilator-associated pneumonia, hospital-acquired infections, nosocomial pathogen, nosocomial infections

## Abstract

Background and objectives: Hospital-acquired pneumonia (HAP) is a life-threatening hospital-acquired infection contributing to poor outcomes and mortality. Though the prevalence is comparable, the burden of comorbidities and malnutrition further worsens the scenario in developing countries. Infective agents responsible for these infections vary between regions due to the variables involved. There is a dearth of data on clinico-microbiological correlates of HAP from Northern India. With this study, we aim to explore the same and add more evidence to fill the gap.

Methodology: A hospital-based cohort study was done on ICU patients of the tertiary care center in Northern India including the cohort of patients obeying a strict inclusion criterion. The clinical and microbiological correlates were estimated following an appraisal of quality of study samples.

Results: We found that the most common clinical feature in patients with HAP was fever (82%) followed by purulent respiratory secretions (72%), tachycardia (52%), and crepitations on auscultation (38%). Approximately 86% of cases were found to be culture-positive while others were bacteriologically sterile. Gram-negative bacilli were more commonly isolated (83% Gram-negative vs 17% Gram-positive). The most common organisms isolated were Klebsiella pneumoniae, Citrobacter freundii, Escherichia coli, Acinetobacter, and Pseudomonas aeruginosa. Staphylococcus aureus was isolated from eight specimens and all isolates were susceptible to vancomycin, linezolid, teicoplanin, and tigecycline. Seven isolates were resistant to clindamycin and all 8 were resistant to macrolides and quinolones. Five strains had methicillin resistance indicating a rising burden of ‘superbugs’. The most common side involved was the right side and the right middle zone was the most common zone involved. Forty-four percent of cases had a poor outcome and succumbed to the infection.

Conclusions: HAP places patients at a heightened risk of mortality and manifests a distinctive clinical-microbiological profile. It is advisable to adopt a proactive stance in averting HAI by adhering to robust prophylaxis and management protocols in alignment with regional data and hospital guidelines. Despite the study's constrained sample size, it contributes significant insights specific to the region. This underscores the necessity for further exploration through analogous studies and audits in the northern part of India. Such endeavors have the potential to tailor treatment approaches for patients, ultimately enhancing overall outcomes.

## Introduction

Hospital-acquired pneumonia (HAP) and ventilator-associated pneumonia (VAP) are the most frequent causes of healthcare-associated infections (HAIs) and represent significant clinical and economic burdens on healthcare systems. HAP and VAP account for approximately 28% of HAIs and are collectively the most frequent cause of nosocomial infections. Epidemiologic data estimate an overall prevalence of nosocomial pneumonia in hospitalized patients of 0.89%, with the burden substantially higher among patients in the intensive care unit (ICU). While the majority of cases of nosocomial pneumonia occur in non-intubated patients, mechanical ventilation is the most important risk factor for pneumonia, increasing the incidence up to 20-fold [1].

The ICU is described as an ‘epidemiological jungle’ because of the abundance of the organisms that proliferate in these units. The predominant organisms responsible for nosocomial infections include *Pseudomonas aeruginosa*, *Staphylococcus aureus, Klebsiella pneumoniae, Escherichia coli,* and other members of* Enterobacteriaceae, Candida *species, etc. [2]. Among nosocomial infections, HAP has the greatest impact on patient management. It is estimated to account for almost half of all ICU infections and accounts for approximately 60% of all deaths from nosocomial infections. Moreover, it increases hospital stays by 7 to 9 days, crude mortality by 70%, and attributable mortality by 30% [3].

HAP and VAP are significant public health issues in Asian countries, as they are worldwide. Worldwide, point-prevalence studies have reported nosocomial infection rates ranging from 6.1% to 15%. Rates seem particularly high in Asia. In a recent study from Malaysia, 14% of hospitalized patients had a nosocomial infection, and 21% of these infections were pneumonia [4].

The epidemiologic data on HAP are scarce from Asia, but various healthcare facilities have reported incidences ranging from 1 to 21 per 1000 hospital admissions. Specific incidences reported to date include 18 per 1000 admissions to the general ward in an Indian study. As would be expected, the incidence of HAP is higher in ICUs. According to various studies in Asian hospitals, the proportion of ICU-acquired respiratory infections ranges from 9% to 23% [5-7].

The literature from Uttarakhand regarding the clinical-microbiological profile of HAP is scarce and limits the personalization of therapy in the region. The study is an attempt to fill in the literature gap. The authors have made an attempt to study the clinical-microbiological profile of patients with HAP and to delineate the cytological features and bacterial etiology of the patients with HAP in Dehradun, Uttarakhand.

## Materials and methods

Study design and study setting

A hospital-based prospective cohort study was conducted in the Department of Medicine, Himalayan Hospital, Swami-Rama Himalayan University, Dehradun and Government Doon Medical College, Dehradun, Uttarakhand, among the patients admitted to the ICU. All the patients with HAP from wards and ICU in the Department of General Medicine were subjected to detailed history and thorough clinical examination according to working proforma. Relevant blood investigations, chest X-rays, and sputum samples of patients were sent for direct smear examination, cytology, and bacteriological culture. 

Case definition

HAP is defined as pneumonia that occurs 48 hours or more after admission, which was not incubating at the time of admission. The diagnosis of HAP is suspected if the patient has a radiographic infiltrate that is new or progressive, along with clinical findings suggesting infection, which include the new onset of fever, purulent sputum, leukocytosis, and decline in oxygenation [8].

Sampling and sample size

A purposive sampling technique was used. All patients admitted to the hospital ICU during the period of six months, who abided by the inclusion criterion, were included in this study. A total of 50 patients were included in the study.

Inclusion and exclusion criteria

All the admitted patients above the age of 18 years who developed new lung infiltrates or had clinical findings suggesting infection two out of three: (a) Fever greater than 38^o^C, (b) purulent respiratory secretions, (c) leukocytosis, or leucopenia were included. The following patients were excluded: (a) Cases of community-acquired pneumonia and (b) patients who were known to be immunosuppressed by human immunodeficiency virus (HIV) infection, under chemotherapy, or post-organ transplant.

Microbiological processing of the aspirate

In the microbiology laboratory, the quality of the sample received was assessed by Gram staining, and if the sample was representative of lower respiratory tract secretion, then it was processed for culture and antibiotic susceptibility.

Quality to access the aspirate

Sputum quality scoring of ET secretion was used (Camargo, 2004) [9]. A smear from the purulent sputum was a Gram-stained smear and observed under a low power (10X) objective. A composite Q score was then calculated depending on the relative number of squamous epithelial cells and the pus cells, as shown in Table 1.

**Table 1:** Composite Q score

Neutrophils				Squamous cells			
	Cells per field			0	1-9	10-24	> 25
		Report		None	Few	Moderate	Many
			Q value	0	-1	-2	-3
	0	None	0	3	0	0	0
	1-9	Few	+1	3	0	0	0
	10-24	Moderate	+2	3	1	0	0
	> 25	Many	+3	3	2	1	0
				Composite Q score			

Sputum quality and culture

Quality scoring (Camargo, 2004) is a very good criterion for assessment. A smear from the purulent part of the sputum is made, and this gram-stained smear was examined under a low (10X) objective.

Interpretation of the Q score

In our study, we employed a scoring system, ranging from Q0 to Q3, to assess the quality of sputum samples obtained. These scores serve as indicators of the degree of representativeness of the samples in relation to upper respiratory tract (URT) and lower respiratory tract (LRT) secretions. A score of Q0 signifies that the sputum sample is not representative of lower respiratory secretions, possibly indicating challenges in obtaining an accurate portrayal of LRT components. Scores of Q1 and Q2 indicate varying degrees of evidence for upper respiratory contamination, with Q1 representing strong evidence (++), and Q2 denoting moderate evidence (+). On the other hand, a score of Q3 suggests that the sample is representative of lower respiratory tract secretions, implying a higher quality in terms of accurately capturing LRT elements. This scoring system aids in the nuanced interpretation of sputum sample quality, shedding light on the potential influences from both upper and lower respiratory regions.

Ethical approval and consent

The study was conducted after ethical approval from the Institutional Ethics Committee of Swami-Rama Himalayan University Hospital with reference number -HIHTPHARMA/I-1/423. Written informed consent from the nearest relative of the patient involved in the study was obtained. The patients and relatives were explained in detail about the risks and benefits of the study and were allowed to opt out of the study at any time before its publication.

Statistical analysis

The data were analyzed qualitatively and quantitatively. The results were expressed as ratios, proportions, and percentages, and relevant statistical tools were applied accordingly.

## Results

Sample characteristics, behavioral patterns, and comorbidities

Out of 50 patients, 32 (64%) were males and 18 (36%) were females. Most patients, 14 (28%), were between the age of 61 - 70 years, while 8 (16%) of patients were above the age of 70 years. The mean age for males was 54.03 years, whereas that of females was 47.05 years. The mean age of the study group was 51.52 years. Twelve patients out of 50 were smokers, while 11 had a history of heavy consumption of alcohol. The most common antibiotics used prior to developing pneumonia were cephalosporins in 25 (50%) of cases, followed by aminoglycosides and metronidazole in 17 (34%) cases. A cerebrovascular accident was the most important associated comorbidity in the patients of the study group. Another common comorbidity was diabetes mellitus in four (8%) patients. Chronic renal failure, coronary artery disease, and chronic obstructive pulmonary disease were seen in two (4%) patients each.

Clinical features

Among the signs in patients with nosocomial pneumonia, fever was the most common sign seen in 41(82%) cases; other common signs were purulent respiratory secretions in 36 (72%) cases, presence of tachycardia in 26 (52%) cases and crepitations on auscultation in 19 (38%) cases (Table 2).

**Table 2:** Signs in patients with nosocomial pneumonia

Clinical signs	Number of patients (%)
Cyanosis	15 (30%)
Fever	41 (82%)
Respiratory secretions	
Mucoid	1 (2%)
Mucopurulent	9 (18%)
Purulent	36 (72%)
Foul smelling	2 (4%)
Serosanguineous	2 (4%)
Heart rate	
< 80	7 (14%)
81- 99	17 (34%)
100 – 119	8 (16%)
120 – 139	16 (32%)
> 140	2 (4%)

Sample quality

Sputum/endotracheal aspirate was obtained from the patients, and the quality of the sample was assessed (Table 3). In our study, we assessed 22 specimens of Q3 quality, which revealed significant growth in 20 (90.9%), whereas out of 22 specimens of Q2 quality, growth was seen in 17 (77.3%).

**Table 3:** Quality of sputum/endotracheal aspirate and results of sputum/endotracheal aspirate culture and blood culture

Quality of sputum	Number of sputum /endotracheal aspirate specimens collected	Sputum /endotracheal aspirate culture		No. of blood specimen collected	Blood culture	
		positive	negative		positive	negative
1	6	6 (100%)	0(0%)	3	2(66.7%)	1(33.3%)
2	22	17(77.3%)	5(10%)	8	5(62.5%)	3(37.5%)
3	22	20(90.9%)	2(9.1%)	11	5(45.5%)	6(54.5%)

Culture and sensitivity

Out of the 50 cases included in the study, 43 (86%) cases were found to be culture-positive, and the remaining 7 (14%) cases were bacteriologically sterile. Gram-negative bacilli were isolated from 39 (83%), while gram-positive cocci were isolated from eight (17%) specimens. The most common organisms isolated were *Klebsiella pneumoniae *and *Citrobacter freundii* which were isolated from 13 (27.7%) specimens, other organisms isolated were *Escherichia coli* in six (12.7%) specimens, *Acinetobacter* in four (8.5%) specimens and *Pseudomonas aeruginosa* in three (6.4%) specimens. *Staphylococcus aureus* was isolated from eight specimens, and all isolates were sensitive to vancomycin, linezolid, teicoplanin, and tigecycline. Seven isolates were resistant to clindamycin and all eight were resistant to macrolides, and quinolones. Five strains were found to be methicillin-resistant Staphylococcus aureus (MRSA) (Table 4).

**Table 4:** Type of bacteria isolated from sputum/endotracheal aspirate culture-positive specimens

Type of bacteria	Type of specimen		Number of isolates
	Sputum	Endotracheal aspirate	
Gram negative bacteria	6	33	39
Acinetobacter	2	2	4
Citrobacter freundii	1	12	13
Escherichia coli	1	5	6
Klebsiella pneumoniae	2	11	13
Pseudomonas aeruginosa	0	3	3
Gram positive bacteria	1	7	8
Staphylococcus aureus	1	7	8

Laboratory findings

Leukocytosis was the most common laboratory finding seen in 46 (92%) cases, which was followed by the presence of neutrophilia seen in 45 (90%) cases (Table 5). Other causes of leukocytosis were ruled out.

**Table 5:** Laboratory findings in the patients of the study group BUN: Blood urea nitrogen; ALT: alanine transaminase; AST: aspartate transaminase

Variable	Number of patients
Hemoglobin ( g/ dl)	
< 10.0	16
10 – 11.9	14
> 12.0	18
Leukocytosis ( / mm^3^ )	
< 10,000	4
10,000 – 19,999	36
>20,000 – 29,999	10
Neutrophilia (%)	
<70	1
70 – 79	4
>80	45
BUN (mg/dl)	
<20	7
20 – 39	33
>40	10
Serum creatinine (mg/dl)	
<1.0	7
1.1 – 2.0	24
>2.0	9
ALT (IU/L)	
<80	40
80 – 160 (2-4X)	6
> 160 (>4X)	4
AST (IU/L)	
<80	42
80 – 160	4
> 160	4
Total bilirubin(mg/dl)	
<2.0	40
2.1 – 3.9	6
>4.0	4

Radiological features

The most common side involved was the right side, and the right middle zone was the most common zone involved. Among 50 cases, 31 cases (62%) cases had more than one zone involvement (Table 6).

**Table 6:** Radiological features of the patients of the study group

Radiological features	Number of patients	%
Type of lesion		
· Bilateral consolidation	16	32
· Left consolidation	7	14
· Right consolidation	17	34
· Left consolidation with pleural effusion	6	12
· Right consolidation with pleural effusion	1	2
· Bilateral patchy infiltrates	3	6
Number of zones involved		
· 1 zone	19	38
· > 1 zone	31	62
Particular zone involved		
· Right upper zone	21	42
· Right middle zone	28	56
· Right lower zone	25	50
· Left upper zone	11	22
· Left middle zone	20	40
· Left lower zone	17	34

Final outcome of the patients

The outcome was known in 43 (86%) cases, out of which 22 (44%) cases had a fatal outcome while 21(42%) cases improved, and seven (14%) patients were lost to follow-up.

## Discussion

The study adds significant data on HAP from a tertiary care center in Northern India. Out of a total of 50 patients in the study group, 64% were males and 36% were females. The mean age for males was 54.03 years, whereas that of females was 47.05 years. The mean age of the study group was 51.52 years. The maximum number of patients 14 (28%) was between the ages of 61 - 70 years, while eight (16%) patients were above the age of 70 years, whereas in a study conducted by Mukhopadhyay et al., the maximum number of patients was between 26 and 50 years [10]. In the present study, the maximum number of patients, 24 (48%), had cerebrovascular accidents, similar to the study by Stebbings et al. where the maximum number of patients had central nervous system disorders [11]. Gupta et al. also reported central nervous system disorders to be the most common clinical finding in patients developing VAP in their study [8].

In our study, fever was the most common sign seen in 41 (82%) cases. Other common features were increased purulence of respiratory secretions seen in 36 (72%) cases. Tachycardia was seen in 26 (52%) of cases, and the presence of crepitations on auscultation was seen in 19 (38%) cases. The Centre for Disease Control and Prevention (CDC) has also included the presence of fever, new onset of purulent sputum, or change in character of sputum as one of the diagnostic criteria of nosocomial pneumonia. The presence of crepitations on auscultation was used to diagnose clinically defined pneumonia. In the laboratory findings of the patients of the study group, leukocytosis was the most common finding seen in 46(92%) cases, which was followed by the presence of neutrophilia, which was seen in 45 (90%) cases. CDC criteria for diagnosis of VAP include leukocytosis as one of the minor criteria [12]. In our study, 41 (82%) patients had early-onset VAP, and early-onset HAP was seen in another five (10%) cases. Endotracheal aspirate (ETA) was the most common sample collected from 46(92%) cases. ETA was preferred over protected specimen brush sampling and bronchoalveolar lavage as these techniques are more invasive, and studies have shown no mortality benefit of using these techniques over ETA. Rajasekhar et al. also reported that the result of ETA was in agreement with bronchoscopy samples [13]. 

The incidence of VAP in a study by Gunalan et al. was high (39.59 episodes per 1000 ventilator days) [14], which was similar to other Indian studies (8.9-46 episodes per 1000 ventilator days) [15]. One possible cause of the high incidence rate may be due to a shortage of nursing staff in the ICU (ideally a 1:1 ratio), which would indirectly affect the care given to the patients. These results were higher when compared with a study done by Elkolaly et al., who found that only 9.6% of patients had early-onset VAP, the possible reason for which could be prior administration of antibiotics [16]. Numerous studies have centered on the causative agents responsible for the development of VAP, and the rate of VAP and its resistance pattern varies depending on the place of study. A survey conducted by Hashemi et al. in Iran showed that 24.6% of VAP was caused by *A. baumannii* followed by *P. aeruginosa* (20.2%) [17].

A similar study by Rocha et al. in Brazil showed that *P. aeruginosa* (29%) was the commonest organism causing VAP, followed by S. aureus (26%) and Acinetobacter spp. (18%) [18]. Hejazi et al. reported that 92.59% had polymicrobial VAP and only 7.41% had monomicrobial growth [19]. An Indian study by Kapaganty and Pilli reported that 67.9% had monomicrobial growth and 32.1% had polymicrobial VAP [20]. A study done by Golia et al. showed that *P. aeruginosa* and *E. coli *were the commonest organisms causing both early- and late-onset VAP [21].

We used the quality score for the assessment of a true sputum specimen, and the composite Q score was calculated depending on the relative number of epithelial cells and the pus cells. In our study, we assessed 22 specimens of Q3 quality, which revealed a significant growth in 20 (90.9%) cases, whereas out of 22 specimens of Q2 quality, growth was seen in 17 (77.3%). Out of the 43 culture-positive cases, 39 (90.7%) cases yielded monomicrobial growth on culture, while 4 (9.3%) yielded two organisms. Rajasekhar et al. found that 81.8% of patients with VAP had an infection with only one type of organism [13]. In our study, Gram-negative bacilli (GNB) were the most common organisms isolated from 83% of specimens. This finding is in concordance with the findings of Estes and Meduri who determined GNB to be the major organism isolated in VAP [22]. In our study, the most common organisms isolated were *Klebsiella pneumoniae* and Citrobacter freundiiwhich were isolated from 13 (27.7%) cases, followed by Staphylococcus aureus seen in eight (17%) cases, other organisms isolated were Escherichia coli in six (12.7%) cases, *Acinetobacter* in four (8.5%) cases, and *Pseudomonas aeruginosa* in three (6.4%) cases. In a similar study by Gupta et al. [8], the most common organism isolated was *Pseudomonas aeruginosa* followed by MRSA, *Klebsiella pneumoniae,* and Acinetobacter. The difference in bacteria isolated from culture shows a significant regional variation and further enforces the need for regional and institutional audits/research to find prevalent patterns helping to guide the empirical therapy before cultures.

In our study, *Staphylococcus aureus* was isolated from eight specimens; all isolates were sensitive to vancomycin, linezolid, teicoplanin, and tigecycline. Seven isolates were resistant to clindamycin, and all 8 were resistant to macrolides and quinolones. Five strains were found to be MRSA. In a multicentered Indian surveillance study, resistance was reported to piperacillin/tazobactam (23%), ciprofloxacin (57%), aminoglycosides (60%-65%), and other β-lactam drugs (60%-70%) in GNB. The indiscriminate use of third-generation cephalosporins has been proposed as a reason for the rise of ESBL-producing strains in India [6]. Furthermore, in our study, the radiological distribution was similar to other reported studies, with the most common side involved being the right side and the right middle zone being the most common zone involved, which may be attributed to the anatomy of the respiratory tract [23].

Our study also reported a mortality burden and poor prognosis produced by the HAP, which forms a major concern globally. We therefore underline the need for regional/institutional research to guide the therapy helping better outcomes.

Limitations

Our study is limited in the aspect of lack of generalizability of findings owing to the cross-sectional study design and limited sample size. Despite these limitations, the study adds valuable data from the region and highlights an important clinical issue. 

## Conclusions

The study found that the most common clinical feature in patients with HAP was fever followed by purulent respiratory secretions, tachycardia, and crepitations on auscultation. The major contributor was Gram-negative bacilli including Klebsiella pneumoniae, Citrobacter freundii, Escherichia coli, Acinetobacter, and Pseudomonas aeruginosa. The sensitivity studies indicated a rising burden of ‘superbugs’ resistant to commonly used antibiotics. HAP exposes the patients to a poor natural history translating to a high burden of mortality and presents a unique clinico-microbiological profile. We recommend taking a proactive approach to prevent HAI and following a good prophylaxis and management protocol as per regional data and hospital protocols. Despite the limited sample size, the study adds important conclusions from the region and warrants further investigation by similar studies and audits in the northern part of India which can help individualize the treatment for patients, helping better outcomes.
